# P-396. PREFER-LA: People with HIV (PWH) in the United States with Prior Adherence Challenges with Oral Antiretroviral Therapy (ART) Prefer Cabotegravir + Rilpivirine Long-Acting (CAB+RPV LA) Therapy after Switch

**DOI:** 10.1093/ofid/ofaf695.613

**Published:** 2026-01-11

**Authors:** Zachary Henry, Stephanie Kirk, Maurice Brownlee, Matthew Herrmann, Sheryl Zayas, Katie L Mycock, Neil Reynolds, Hannah Wallis, Mona Amet, Ann Linskey, Jimena Patarroyo, Deanna Merrill, Edgar T Overton, Cindy Garris, Andrew P Brogan

**Affiliations:** AIDS Healthcare Foundation– Northpoint, Fort Lauderdale, FL, Fort Lauderdale, Florida; Medical University of South Carolina, Charleston, South Carolina; Baal Perazim Wellness & Health Services Inc., Chicago, Illinois; Harbor-UCLA Medical Center, Sherman Oaks, California; Care Resource, Miami, FL, Miami, Florida; Adelphi Real World, Bollington, England, United Kingdom; Adelphi Real World, Bollington, England, United Kingdom; Adelphi Real World, Bollington, England, United Kingdom; Adelphi Real World, Bollington, England, United Kingdom; ViiV Healthcare, Durham, North Carolina; ViiV Healthcare, Durham, North Carolina; ViiV Healthcare, Durham, North Carolina; ViiV Healthcare, Durham, North Carolina; ViiV Healthcare, Durham, North Carolina; ViiV Healthcare, Durham, North Carolina

## Abstract

**Background:**

The LATITUDE trial demonstrated that cabotegravir + rilpivirine long-acting (CAB+RPV LA) is superior in efficacy compared to daily oral antiretroviral therapy (ART) in people with HIV (PWH) with documented prior adherence challenges. The real-world experiences of PWH from PREFER-LA (Perspectives on Treatment with CAB+RPV LA Injectable Therapy from PWH in the US with Prior Adherence Challenges to Oral ART) are presented.

**Figure 1.**
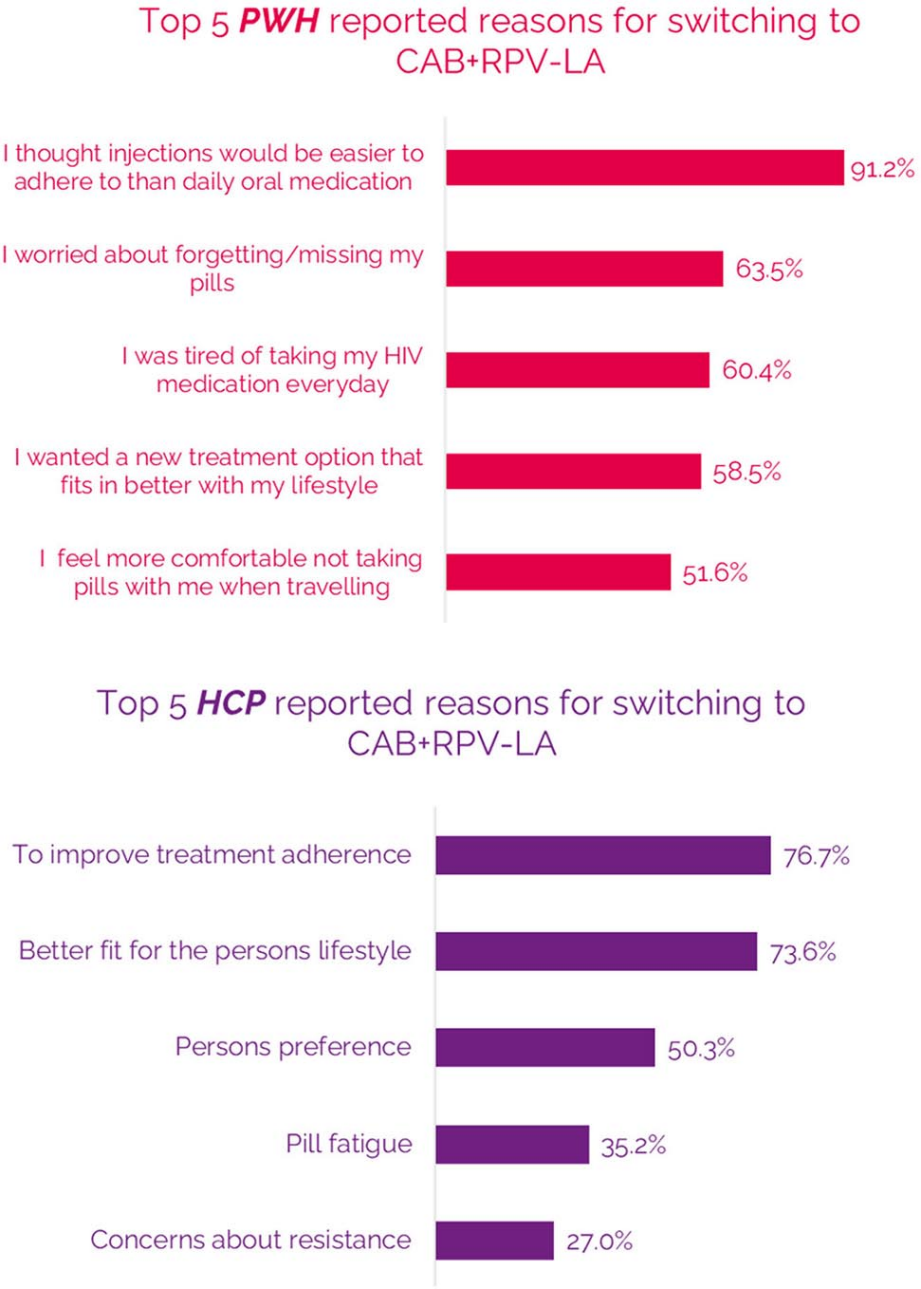
PWH (survey) and HCP (eCRF) reasons for switching to CAB+RPV-LA (n = 159, multiple choices allowed)

**Figure 2.**
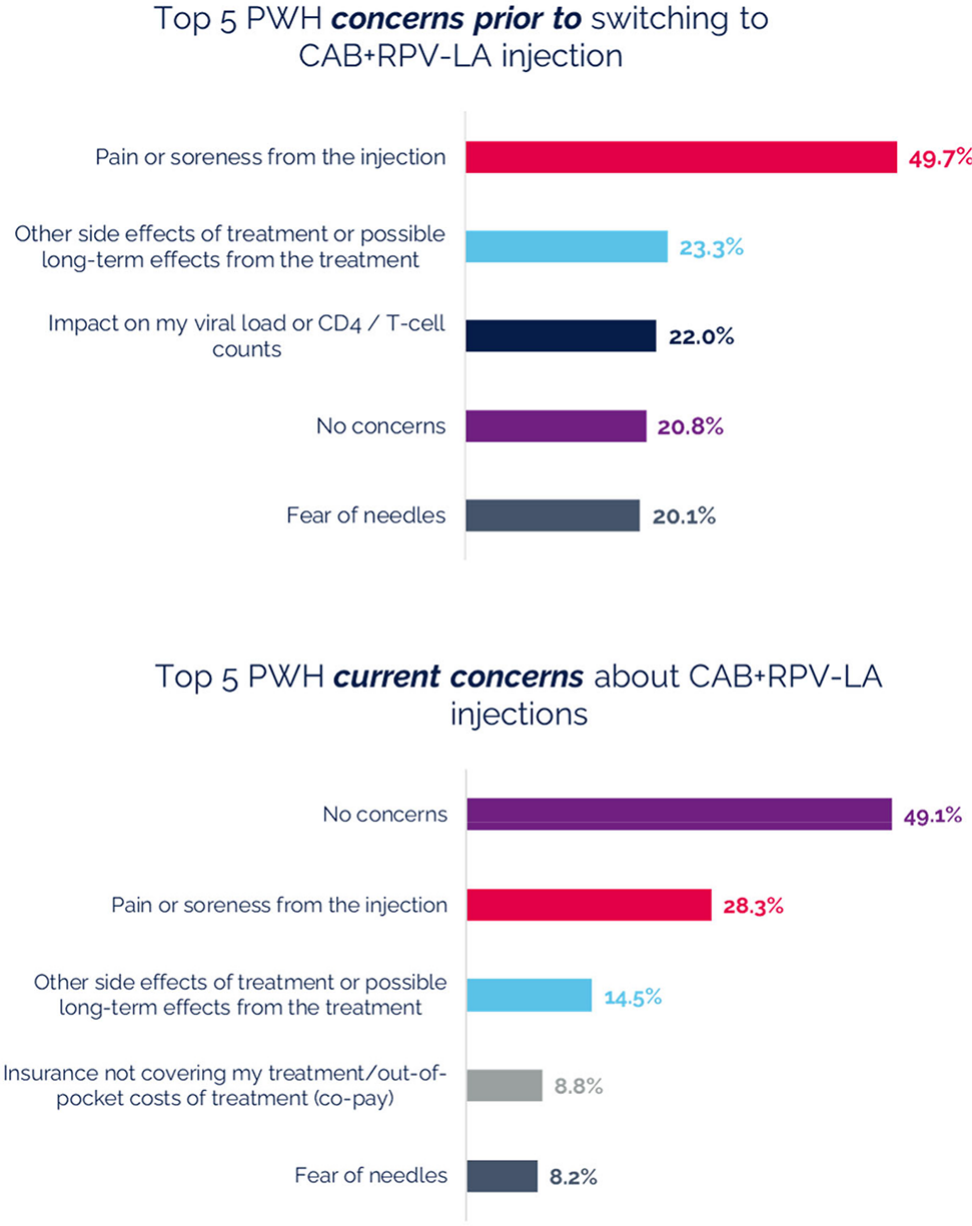
PWH concerns before and after switching to CAB+RPV-LA (PWH survey, n = 159, multiple choices allowed)

**Methods:**

PREFER-LA was an observational real-world study of PWH from across the US receiving CAB+RPV LA for ≥6 months to ≤18 months with documented adherence challenges to prior oral ART. The study consisted of: (1) cross-sectional survey of PWH to evaluate experiences of historical oral ART use and perspectives of treatment with CAB+RPV LA, (2) corresponding retrospective medical chart review (eCRF) to establish treatment history and clinical outcomes, and (3) cross-sectional survey of healthcare providers (HCP) from each site.

**Figure 3.**
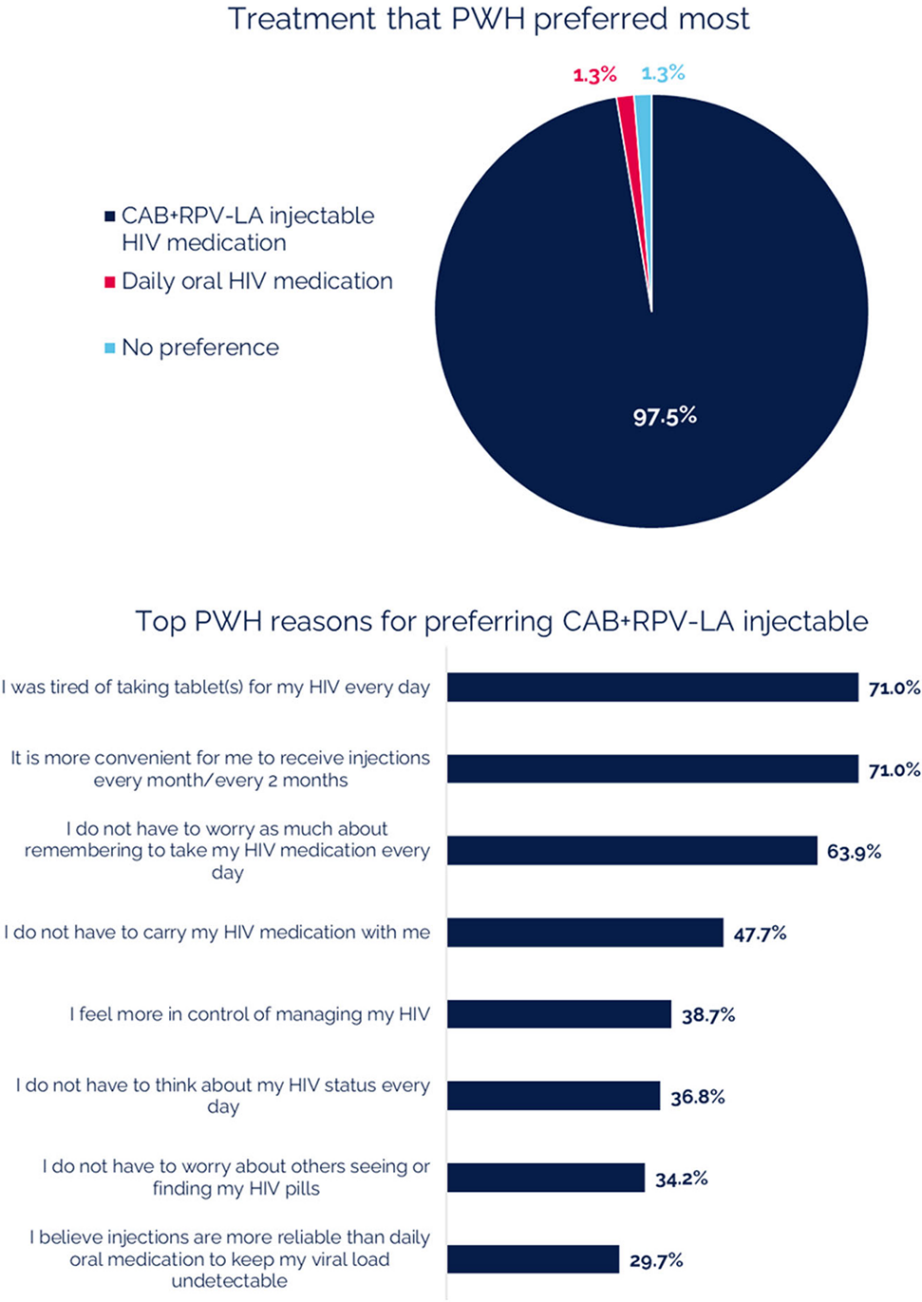
Treatment Preference (PWH survey, n=159)

**Figure 4.**
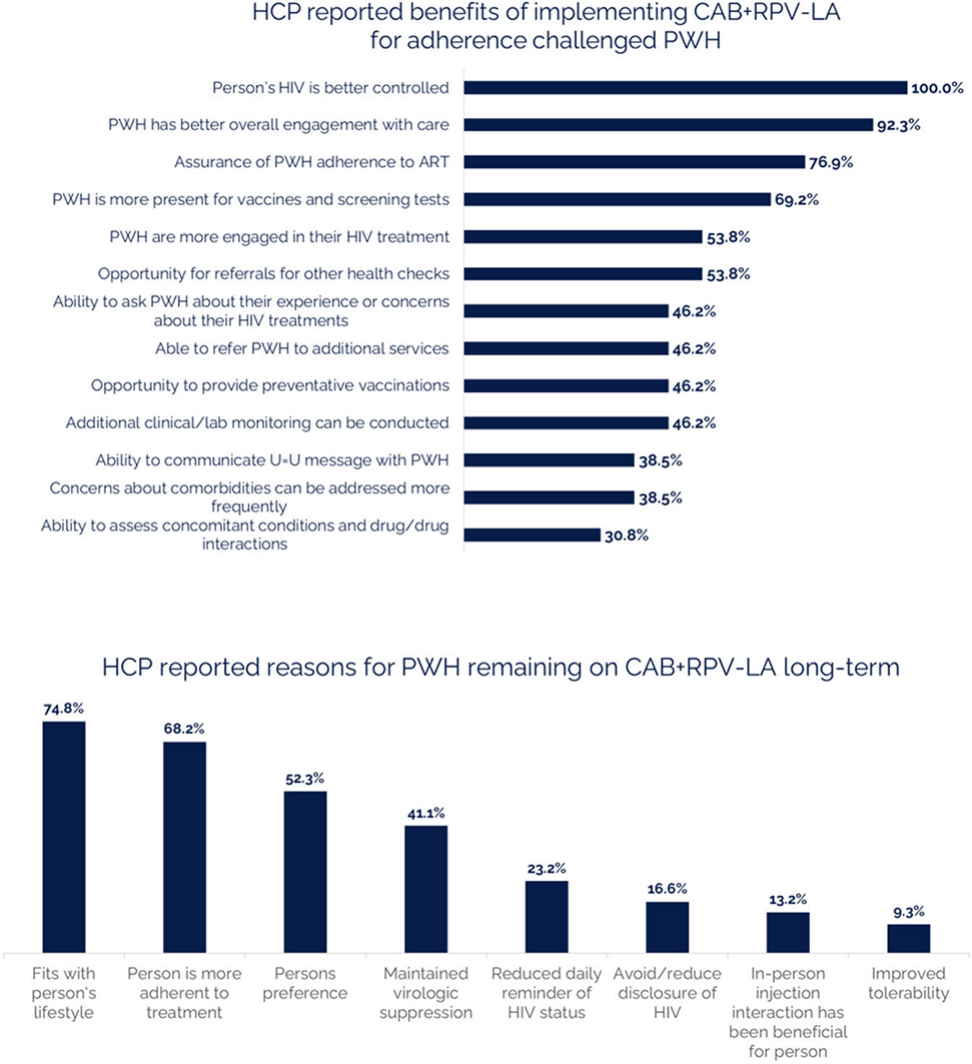
HCP reported benefits of implementing CAB+RPV-LA for adherence challenged PWH and reasons why HCPs think PWH will remain on CAB+RPV-LA long-term (HCP survey, n=13)

**Results:**

Median age of participants (n=159) was 39 years old and median time since HIV diagnosis was 11.7 years. Majority were Black/African American (57%). The primary driver for switching to CAB+RPV LA for both PWH and HCP focused on adherence (Figure 1). Most PWH concerns about switching to a long-acting injectable were alleviated after switching to CAB+RPV LA (Figure 2). The majority of PWH agreed that switching to CAB+RPV LA has positively affected their HIV control (79%), their overall health (67%), quality of life (79%) and better fits their lifestyle (83%). When prompted, PWH reported more positive feelings towards themselves (79%) since switching to CAB+RPV LA and 90% of PWH were very likely to recommend CAB+RPV LA to other PWH with adherence challenges. Almost all PWH preferred CAB+RPV LA over daily oral ART (Figure 3). HCPs reported several benefits for CAB+RPV LA (Figure 4) and that 95% of PWH would remain on CAB+RPV LA long term. Most PWH (77%) reported benefits from regular clinic visits, including ensured adherence, HIV control, and more opportunities to discuss health concerns.

**Conclusion:**

In this observational real-world study, PWH switching to CAB+RPV LA after experiencing prior adherence challenges on oral ART overwhelmingly preferred CAB+RPV LA due to numerous benefits of switching to a long-acting injectable.

**Disclosures:**

Zachary Henry, DO, gilead: Grant/Research Support|Therapharmaceutical: Grant/Research Support|ViiV Healthcare: Grant/Research Support Stephanie Kirk, PharmD, ViiV Healthcare: Grant/Research Support Maurice Brownlee, APRN, EMD Serono: Grant/Research Support|EMD Serono: Honoraria|Gilead Sciences: Grant/Research Support|Gilead Sciences: Honoraria|Napo Pharmaceuticals: Grant/Research Support|Napo Pharmaceuticals: Honoraria|ViiV Healthcare: Grant/Research Support|ViiV Healthcare: Honoraria Neil Reynolds, PhD, Adelphi Real World: Advisor/Consultant Hannah Wallis, MS, Adelphi Real World: Advisor/Consultant Mona Amet, MPH, Adelphi Real World: Advisor/Consultant Ann Linskey, PharmD, AAHIVP, ViiV Healthcare: employee|ViiV Healthcare: Stocks/Bonds (Public Company) Jimena Patarroyo, PharmD, AAHIVP, ViiV Healthcare: Stocks/Bonds (Private Company) Deanna Merrill, PharmD, MBA, AAHIVP, ViiV Healthcare: employee Edgar T. Overton, MD, ViiV Healthcare: Employment|ViiV Healthcare: Stocks/Bonds (Public Company) Cindy Garris, MS, ViiV Healthcare: employee|ViiV Healthcare: Stocks/Bonds (Public Company) Andrew P. Brogan, PhD, ViiV Healthcare: Employee|ViiV Healthcare: Stocks/Bonds (Public Company)

